# Impact of Immunoglobulin M-Type Donor-Specific Human Leukocyte Antigen–Antibody Levels in Supernatants from Cultured Peripheral Blood Mononuclear Cells as Predictors of Antibody-Mediated Rejection

**DOI:** 10.3390/pathogens9090733

**Published:** 2020-09-05

**Authors:** Ryoichi Imamura, Yoshiko Matsuda, Koichi Tsutahara, Norio Nonomura, Shiro Takahara

**Affiliations:** 1 Department of Urology, Osaka University Graduate School of Medicine, Osaka 565-0871, Japan; imamura@uro.med.osaka-u.ac.jp (R.I.); nono@uro.med.osaka-u.ac.jp (N.N.); 2 Department of Advanced Technology for Transplantation, Osaka University Graduate School of Medicine, Osaka 565-0871, Japan; 3 Department of Urology, Osaka General Medical Center, Osaka 558-8558, Japan; k.tsuta@gmail.com; 4 Department of Renal Transplantation, Kansai Medical Hospital, Osaka 560-0083, Japan; shiroutakahara@gmail.com

**Keywords:** antibody-mediated rejection, kidney transplantation, donor-specific anti-HLA antibody, IgM antibody, IgM memory B cell, in vitro culture assay

## Abstract

Background: Antibody-mediated rejection (AMR) is a crucial barrier in the long-term prognosis of transplant recipients. Methods: Peripheral blood mononuclear cells (PBMCs) were collected from kidney allograft recipients (*N* = 41) and cultured in vitro for 1 week. Furthermore, the supernatants of the cultured PBMCs were analyzed by Luminex single-antigen beads. Results: Analyses using Luminex single-antigen beads revealed the presence of immunoglobulin (Ig) G donor-specific anti-HLA antibodies (DSAs) was detected in the supernatants of cultured PBMCs collected more frequently than IgM in *de novo* DSA-sensitized patients with AMR, and IgM were detectable in patients with stable graft function mainly and several IgM DSAs were detectable in the supernatants of the cultured PBMCs before detecting the IgG levels in sera. We also found that the DSA-specific IgM-secreting memory B cells (mBCs) were more sensitive to the chronic use of immunosuppressive agents than to the IgG-secreting mBCs. Conclusions: In the transplant recipients, the assessment of supernatants of cultured PBMCs provide more details of immune reactions than the commonly used method that directly measures IgG DSA levels in patient sera and some IgM DSA detection may be a better predictor of IgG DSAs production, which may cause AMR and enable early intervention, in initial stages of AMR development.

## 1. Introduction

Anti-human leukocyte antigen (HLA) donor-specific anti-HLA antibodies (DSAs) play important roles in the development of antibody-mediated rejection (AMR) of transplanted biomaterials [1]. Technological improvements have enabled the detection of increasingly lower anti-HLA antibody (Ab) levels in patient sera, thereby remarkably decreasing the frequency of hyperacute rejection by preformed DSAs [2,3]. Thus, the identification of DSAs prior to a transplant could help in guiding the use of desensitization therapies; however, to date, early detection of the generation of *de novo* DSAs in serum, which are produced after transplantation, is difficult, thereby thwarting early treatment interventions to prevent the development of AMR.

DSAs are produced from the plasma cells (PCs) via the recognition of donor-specific antigens by antigen-presenting cells, which are utilized by naïve B cells, processed, and are presented on the surface of the cells where they bind to the antigen-specific T-cell receptors. The activated T cells then promote B-cell proliferation, immunoglobulin (Ig) class-switching, gene conversion, and enhanced antigen affinity via VDJ (variable, diversity, and joining) recombination through the germinal center (GC). In addition, the macrophages and dendritic cells activated by the effector T cells produce cytokines, which aid in B-cell proliferation and differentiation. Subsequently, the selected memory B cells (mBCs), which have a high affinity for donor-specific antigens, differentiate into PCs [4,5]. It has been proposed that by the time DSAs are detected in the sera by binding to the donor-specific antigens, the graft already sustains injury [6].

Furthermore, the chronic use of immunosuppressive agents to repress T-cell proliferation may not significantly inhibit PC survival, because PCs may survive in a T cell-independent manner [7]. More recently, efficient immunosuppressive therapies have been considered necessary in inhibiting PC growth and survival; however, such interventions are, in general, accompanied by severe side effects, such as pancytopenia, anemia, and viral infection [8,9]. Thus, the early detection of DSA-secreting mBCs may allow for timely intervention for AMR control [10,11]. Hence, the present study aimed to investigate the possibility that by evaluating the mBC-derived Abs in the supernatant of cultures peripheral blood mononuclear cells (PBMCs), IgM DSAs might be useful as early diagnostic markers to provide additional information about the development of AMR.

## 2. Results

### 2.1. Participants Characteristics

This study followed the principles of the Declaration of Helsinki, and all subjects provided informed consent to participate. The Osaka university ethics committee has approved this study. Peripheral blood (8 mL) and serum were collected from 41 kidney allograft recipients (20 males and 19 females; age range: 6–77 years; average, 44.2 ± 16.0 years) before and after transplants, which had been performed in Osaka University Hospital between January 2000 and January 2017. Table 1 showed the baseline characteristics in 41 allograft recipients.

### 2.2. Isotypes (IgG or IgM) of DSAs Produced from PBMC Culture Supernatants Reflect the Activation Level of Humoral Immune Responses against Donor-Specific Antigen; Several IgM DSAs May Be a Precursor of the Production of IgG DSAs in the Patient Sera

**Figure 1:**
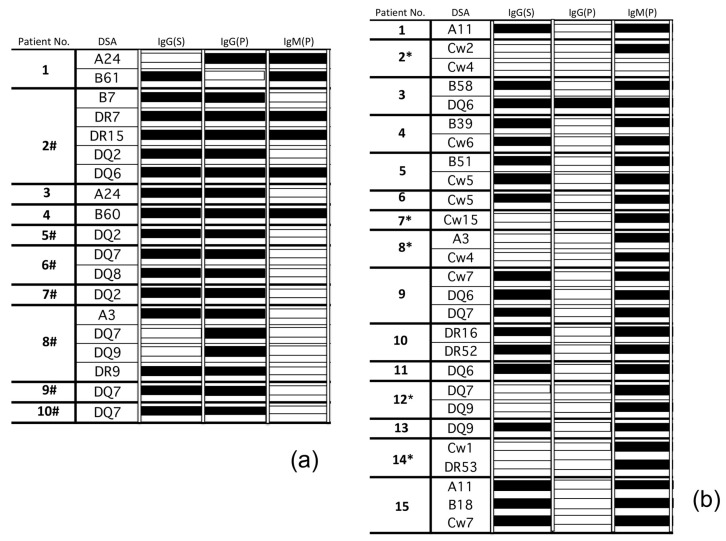
Peripheral blood mononuclear cells (PBMCs) were collected from the kidney allograft recipients, and then cultured. For only FlowPRA screening positive cases, LABScreen Single Antigen HLA Class I and/or Class II (One Lambda, Canoga Park, CA, USA) was used to analyze antigen specificity and shown. (**a**) IgG DSAs were detectable in the PBMC cultured supernatants (*N* = 10). Antibody contents of the supernatants and serum were then analyzed using Luminex single-antigen beads. Data are presented from 10 independent experiments. (**b**) IgM DSAs were detectable in the PBMC cultured supernatants (*N* = 15). Antibody contents of the supernatants and serum were then analyzed using Luminex single-antigen beads. Data are presented from 15 independent experiments. Filled bars represent the detection of antibodies by Luminex single-antigen beads and open bars represent the absence of antibodies. At the time of blood collection, some cases were diagnosed with AMR (#) and IgG DSAs were not detected from the serum (*). *S*, serum; *P*, PBMC culture supernatant.

PBMCs were obtained from allograft recipients who were sensitized to *de novo* DSAs and then cultured in vitro. Whereas Luminex single-antigen beads successfully detected IgG DSAs in cultured supernatants of PBMCs more frequently than IgM DSAs in *de novo* DSA-sensitized patients with AMR (Figure 1a). In the subsequent assays, IgM DSAs were mainly detectable in the supernatants of cultured PBMCs from *de novo* DSA-sensitized patients with a stable graft function. However, several IgM DSAs were detected in the supernatants of cultured PBMCs from non DSA-sensitized patients whose IgG DSAs were not detected from the serum (*; Figure 1b).

**Figure 2:**
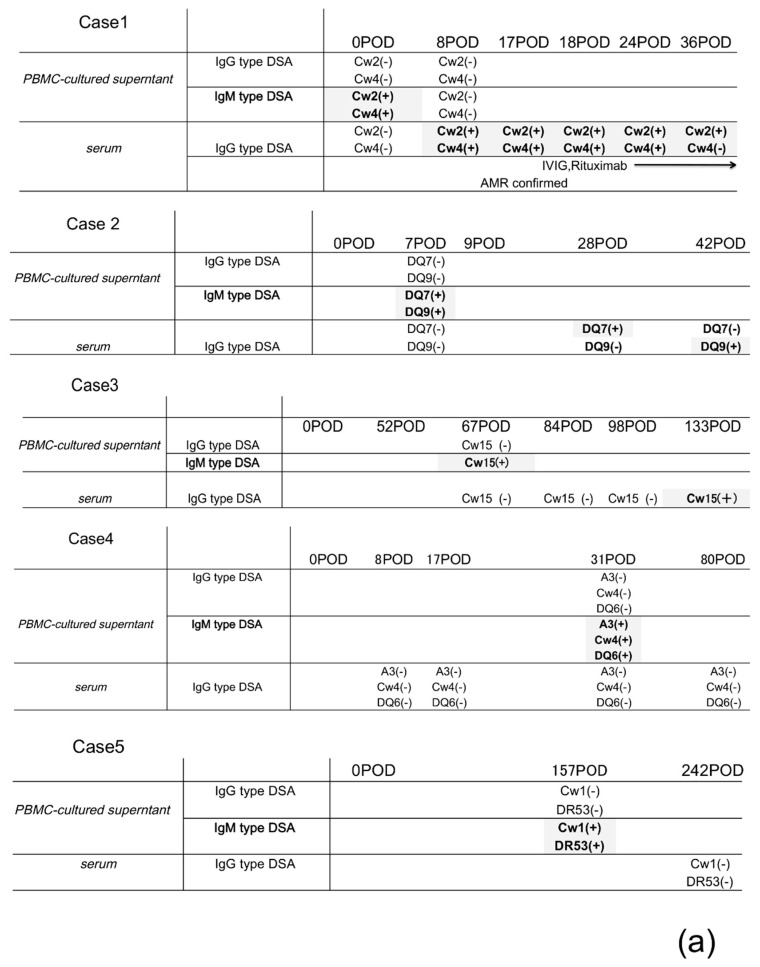

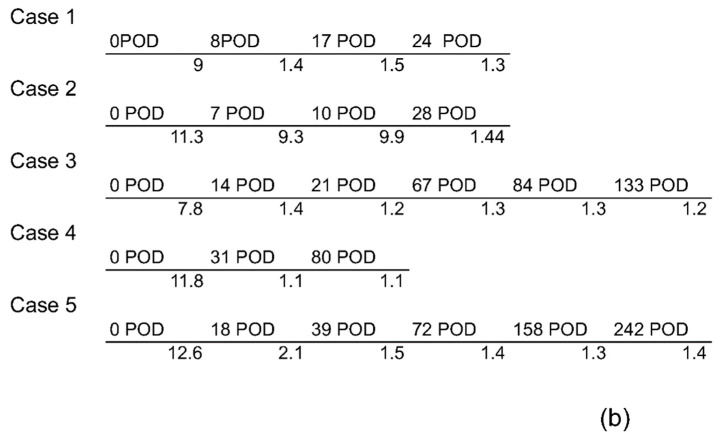
(**a**) IgG and IgM DSAs from PBMC supernatants and IgG DSAs from serum and kidney biopsies were determined during the clinical course and are summarized in a time series (*N* = 6). HLA was considered positive when it was detected at an MFI of 250 or higher. Serum and PBMC were collected every week for up to 1 month after transplantation and every 2−3 months thereafter. POD, postoperative day. The results of the day IgG/IgM DSA was detected in the PBMC culture supernatant or serum, the date of any event such as rejection, and the date of irregular blood sampling were indicated. PBMC supernatants and serum were analyzed by Luminex single-antigen beads evaluation, and we described whether IgG/IgM DSAs were detectable (+) or undetectable (–) in (**a**). (**b**) The creatinine changes (mg/dL) in the five cases shown in (**a**) are in a time series.

**Figure 3:**
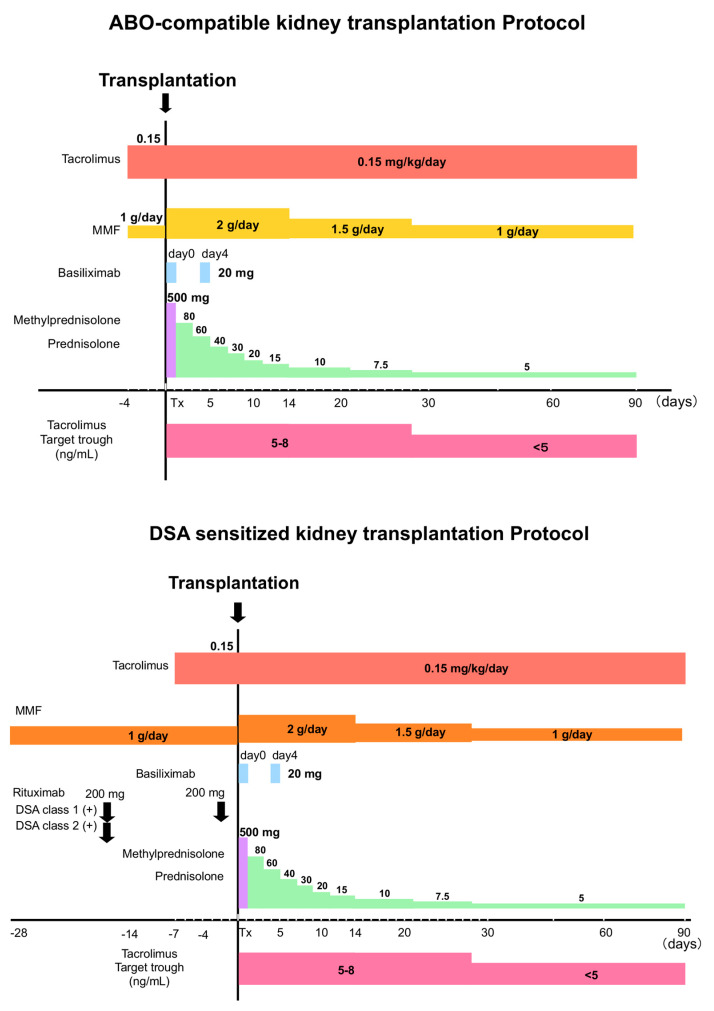
Protocolized immunosuppression for kidney transplantations in our institute was summarized.

Thus, IgG and IgM DSAs from PBMC supernatants and IgG DSAs from serum and kidney biopsies were determined during the clinical course and are summarized in a time series (Figure 2a) and patients were treated following protocolized immunosuppression in our institute (Figure 3).

In several patients (cases #1–3), certain IgM DSAs were detected in the cultured supernatants before IgG DSAs being detectable in cultured supernatants or sera. In cases #4 and #5, IgG DSAs were not detected in the cultured supernatants or serum after the detection of IgM DSAs within an observation. In several patients (cases #1–3), certain IgM DSAs were detected in the cultured supernatants before IgG DSAs being detectable in cultured supernatants or sera. In cases #4 and #5, IgG DSAs were not detected in the cultured supernatants or serum after the detection of IgM DSAs within an observation.

### 2.3. IgM-Secreting mBCs Are More Sensitive to the Standard Immunosuppressive Therapy Than IgG-Secreting mBCs

**Figure 4:**
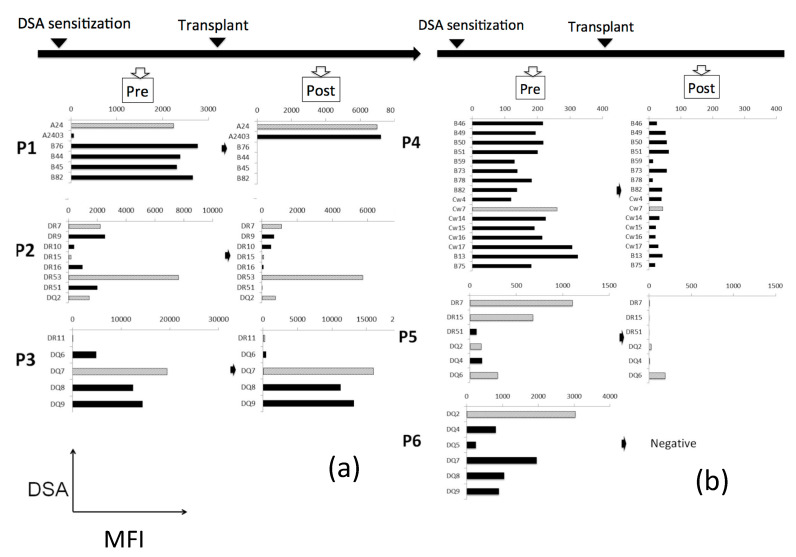
PBMCs were isolated and cultured from the secondary kidney allograft recipients who were sensitized to the primary donor-specific antigen. During secondary transplantation, the recipients received induction therapy and standard immunosuppressive agents. (**a**) IgG Abs against primary donor-specific antigens were detected from the PBMC culture supernatants before secondary transplantation (Patients 1, 2, and 3). Levels of IgG Abs against the primary donor-specific antigens were compared between pre- and post-secondary transplantation. Data are presented from three independent experiments. (**b**) IgM Abs against the primary donor-specific antigens were detected from the PBMC culture supernatant before secondary transplantation (Patients 4, 5, and 6). Levels of IgM Abs against primary donor-specific antigens were compared between pre- and postsecondary transplantation. Data are presented from three independent experiments. The closed bar indicates DSA and the hatched bar indicates non-DSA. “Negative” indicates that HLA antibodies were not detected by Flow PRA screening and the supernatant was not analyzed by Luminex single-antigen bead evaluation.

PBMCs were collected from the secondary transplant recipients (*N* = 6) after sensitization to the primary donor antigens and were then cultured in vitro. Subsequently, the supernatants from these cultures were assayed before and after transplantation, and recipients were then treated with rituximab (monoclonal anti-CD20 antibody) as an induction therapy, followed by chronic use of the immunosuppressive agents prednisolone, tacrolimus, and mycophenolate. In the ensuing analyses, almost all IgGs against the primary donor antigens were detected at similar or higher levels at both time points (Figure 4a); however, the levels of almost all IgMs against the primary donor antigens were decreased remarkably after the second transplant (Figure 4b).

## 3. Discussion

AMR of transplants leads to poor long-term prognoses; however, it cannot be controlled due to the lack of effective early diagnostic methods other than tissue biopsies and the related pathological status had progressed irreversibly at the time of diagnosis. Moreover, DSAs are associated with AMR [12,13] and thus, are considered as useful biomarkers for AMR development. Unfortunately, many reports indicated that DSAs might bind to the graft and only a few “overflow” antibodies are left undetected, rendering problematic detection, in particular, during the early phases of AMR [14]. Therefore, the clinical potential of DSA-specific circulating mBCs in the peripheral blood has recently attracted attention as an earlier diagnostic method.

In some previous reports, the frequency of DSA-specific mBCs circulating in the peripheral blood was evaluated by enzyme-linked immunospot (ELISPOT) analyses, which revealed that a high frequency of DSA-specific mBCs in the peripheral blood was associated with greater injury to the kidney grafts and patient history of AMR [10,11,15]. Therefore, ELISPOT assays of these cells in the peripheral blood may facilitate the monitoring of humoral immune reactions against donor-specific antigens.

Alternatively, some researchers attempted to induce mBCs differentiation into PCs in vitro and analyze mBCs-derived Abs from the cultured supernatants. In these studies, CD19^+^ B cells were cultured on CD40 ligand feeder cells (EL4-B5) with growth factors and cytokines from activated T cells [16,17,18]. Nevertheless, B cells are sensitive to the separation process, and direct support from the helper T cells in a cell–cell contact manner is crucial in the growth and survival of mBCs [18,19,20,21,22]. Accordingly, we attempted to establish a more physiologically accurate in vitro assay to culture PBMCs in order to induce mBC differentiation into PCs by maintaining cell–cell interactions between mBCs and helper T cells (including interactions via the CD40 ligand), macrophages, and dendritic cells [23,24]. It has also been reported that natural killer (NK) cells or monocytes suppress the growth and survival of mBCs in vivo through the suppression of helper T-cell activation and production of cytokines involved in the maintenance of B cell survival or B cell apoptosis, among others, and those accessory cells are involved in the immunoregulatory mechanism [25,26].

However, in our culture system, phorbol myristate acetate (PMA) may be involved in inhibiting NK cell differentiation and activation [27], phytohemagglutinin/leucoagglutinin (PHA-L) stimulates T cells in a lectin extract that binds to T cell membranes and subsequently the production of proinflammatory cytokine is promoted from the monocytes by stimulated T cells in a cell–cell contact manner [28]. Consequently, there is a possibility that the inhibitory effect on mBC activation may be suppressed. In other words, our culture system may be suitable for the differentiation of mBCs corresponding donor-specific HLA into PCs. However, considering the involvement of those accessory cells described above, it is not yet clear whether PCs are actually induced to differentiate in vivo like in vitro. Therefore, in the future, it is necessary to examine in detail the environmental factors or inhibitory factors that induce mBCs corresponding donor-specific HLA to differentiate into PCs and these findings may lead to the development of new effective therapeutic methods for the prevention of AMR.

Considering the evaluation of DSA isotypes, IgG DSAs have gained attention in the field of transplantation mainly due to their antigen specificity. By contrast, IgM DSAs have been considered to be unreliable markers, even though some IgM mBCs activate T cells dependent on reactions in the GCs and produce IgM Abs with high affinity for antigens [29]. Certain reports indicated a possibility that natural IgM Abs, which account for the majority of serum IgM, may bind to beads as anti-HLA Abs due to the cross-reactivity or inhibition of antigen-specific Ab detection by beads [30,31,32,33]; however, a previous study by our group found that PBMC culture analysis was less susceptible to natural Abs than serum [34].

In the present experiments using Luminex single-antigen beads, we evaluated the production of IgG and IgM DSAs in the cultured PBMCs from allograft recipients who were sensitized to *de novo* DSAs. Our findings suggested that IgG DSAs were mainly present in the culture supernatants of PBMCs from *de novo* DSA-sensitized patients with confirmed AMR, which represented the activation of humoral immunity against donor-specific antigens.

In contrast, IgM DSAs were detected mainly in the supernatant of cultured PBMCs collected from *de novo* DSA-sensitized patients with stable graft function (Figure 1b). Thus, assessment of both IgG and IgM DSA levels in the supernatant of cultured PBMCs might reflect the activation level of humoral immunity against the donor-specific antigens more accurately than IgG from serum.

IgG DSA detection in sera revealed that PCs in the bone marrow may continue producing IgG DSAs, regardless of the activation level of the humoral immunity against the donor-specific antigens for a long term after sensitization to these antigens; however, in several cases, IgM DSAs were detected in the PBMC supernatants when IgG DSAs were not detected in serum; thus, we compared the IgG/IgM DSA detection in the supernatants of cultured PBMCs with serum IgG levels and the clinical course of the disease over time. The results revealed that several IgM DSAs were detectable in the supernatant of cultured PBMCs before IgG detection in serum (Figure 2a).

In cases #1 and #2, IgM DSAs were produced in the cultured supernatants early after transplantation (days 0 and 8) and IgG DSAs were detected in sera soon afterwards. In case #2, AMR was confirmed in the biopsy samples 9 days after detecting IgG DSAs in the serum.

While detecting IgM DSA in the supernatant of the cultured PBMCs and IgG in sera, the DSA-specific IgM mBCs may grow and class-switch to IgG-.

In cases #3, #4, and #5, IgM DSAs were produced in the cultured supernatants from one to a few months after transplantation (days 31, 67, and 157, respectively); however, several months had lapsed between the detection of IgM DSA in supernatants and detection of IgG in serum or IgG was not detected in serum within this observation.

In addition, we reported previously that activation of the antigen–antibody reactions through B-cell receptors might mediate class-switching of IgM to IgG and that the production of growth factors and cytokines by activated T cells, macrophages, and dendritic cells promoted this process [34]. Thus, it is possible that the rapid reduction in immunosuppression (induction therapy or chronic use of immunosuppressive agents) might promote the growth of DSA-specific IgM mBCs or class-switching to IgG, as the immunosuppression of active T cells and inflammatory cells might be reduced rapidly early after transplantation (Figure 3); however, even with stabilized immunosuppressive therapy, we confirmed the detection of IgG DSAs in serum after IgM detection in the supernatant of cultured PBMCs in one of these three cases. Accordingly, other factors may be involved in the growth and survival of IgM mBCs and class-switching of IgM to IgG, other than reduction of immunosuppressive agents. In children, the *de novo* DSA production and the probability of graft loss due to *de novo* DSA are higher than those in adults [35], there may be differences in the immune system between children and adults. Besides these problems, there are also adherence issues in pediatric cases. Accordingly, it is deemed necessary to compare the factors that induce IgM differentiation and class switching in children and adult cases in larger cohorts and take measures to prevent AMR development according to the age.

In further experiments, we examined the differences in responsiveness to the immunosuppressive agents between DSA-specific IgG mBCs and circulating IgM levels in peripheral blood. In these experiments, PBMCs were collected from secondary kidney recipients who were sensitized using primary donor antigens, and IgG and IgM levels against primary donor-specific antigens were compared before and after secondary transplantation. Induction therapy to inhibit T-cell survival and chronic use of immunosuppressive agents were administered at the time of secondary transplantation.

Subsequent analyses revealed that IgG DSA levels in the supernatants of cultured PBMCs were maintained after induction therapy, whereas IgM DSAs were remarkably reduced (Figure 4). These data indicate that more severe treatment is required to remove the DSA-specific IgG mBCs, and DSA-specific IgM mBCs are more sensitive to the less invasive immunosuppressive agents than to IgG mBCs.

## 4. Materials and Methods

### 4.1. Isolation and Culture of PBMCs

PBMCs were isolated using a Ficoll-Hypaque density gradient (Sigma–Aldrich, St. Louis, MO, USA). The cells were cultured in 24-well flat-bottomed plates (5 × 10^5^ cells/well) in basal B-cell culture medium composed of Iscove’s modified Dulbecco’s medium (Sigma–Aldrich) with 10% fetal calf serum (Thermo Scientific HyClone, Logan, UT, USA) supplemented with 50 µg/mL human transferrin–selenium and 5 µg/mL human insulin (Gibco/Invitrogen Co., Carlsbad, CA, USA) in the presence of the molecules as follows: 50 ng/mL interleukin (IL)-21, 2.5 µg/mL phosphorothioate CpG-oligodeoxynucleotides (ODN) 2006, 2.5 µg/mL phytohemagglutinin/leucoagglutinin (PHA-L), and 15 ng/mL phorbol myristate acetate (PMA).

### 4.2. Reagents

Recombinant human IL-21 was purchased from Miltenyi Biotech. Phosphorothioate CpG-ODN 2006 was purchased from Invivogen (San Diego, CA, USA). PHA-L and PMA were purchased from Sigma–Aldrich (St. Louis, MO, USA).

### 4.3. Detection of HLA Antibodies in the Culture Supernatants

We collected PBMC culture supernatants and concentrated these by 5-fold prior to the FlowPRA screening test based on Flow PRA 60 kits (One Lambda, Canoga Park, CA, USA). For only positive cases, LABScreen Single Antigen HLA Class I and/or Class II (One Lambda, Canoga Park, CA, USA) was used to analyze antigen specificity.

### 4.4. Statistical Analysis

All data are expressed as median values and ranges unless otherwise indicated. An analysis of variance (ANOVA) or a paired or unpaired Student’s *t* test was used to evaluate the significance of differences between variables. Prism 7 software (GraphPad Inc., San Diego, CA, USA) was used for statistical analyses. A two-tailed *p* value < 0.05 was considered statistically significant.

## 5. Conclusions

The assessment of IgM DSAs levels in cultured PBMCs may be employed as a biomarker, which may provide an opportunity for early interventions before IgG DSAs production, which may result in AMR development in serum by using 8 mL of peripheral blood, after determining the characteristics of DSA-specific IgM mBCs and subsequent class-switching to IgG. Moreover, risk factors promoting IgM class-switching to IgG and the time required for DSA-specific IgM mBC to differentiate into IgG PCs warrant further investigation in a larger cohort for clinical application.

## Figures and Tables

**Table 1:** An analysis of variance (ANOVA) was used to evaluate the significant differences between four groups: graft function stable, antibody-mediated rejection (AMR) confirmed, non-donor-specific anti-HLA antibody (DSA) sensitized, and HLA non-sensitized. A two-tailed *p*-value < 0.05 was considered statistically significant. Baseline characteristics (*N* = 39).

Parameter		Graft function Stable (*N* = 10)	AMR Confirmed (*N* = 7)	Non-DSA Sensitized (*N* = 12)	HLA Non-Sensitized (*N* = 10)	*p-*Value
Gender (Male/Female)	*N* (%)	4(40%)/6(60%)	5(71.4%)/2(28.6%)	6(50%)/6(50%)	5(50%)/5(50%)	N.S
Age at transplant (Year)	Mean ± SD	48.8 ± 9.6	41.6 ± 19.0	49.8 ± 11.4	36.8 ± 15.9	N.S
ABO compatibility (Compatible/Incompatible)	*N* (%)	10(100%)/0(0%)	7(100%)/0(0%)	11(91.7%)/1(8.3%)	9(90.0%)/1(10%)	N.S
Immunosuppression						
Maint. Pred (Yes/No)	*N* (%)	10(100%)/0(0%)	7(100%)/0(0%)	11(91.7%)/1(8.3%)	7(70%)/3(30%)	N.S
1 = Tac, 2 = CyA	*N* (%)	10(100%)/0(0%)	7(100%)/0(0%)	12(100%)/0(0%)	10/(100%)/0(0%)	N.S
1 = MMF, 2 = MZ	*N* (%)	10(100%)/0(0%)	7(100%)/0(0%)	12(100%)/0(0%)	10/(100%)/0(0%)	N.S
Kidney biopsy						
g + ptc	Mean ± SD	0.83 ± 0.69	3.5 ± 1.0			<0.001 ***
C4d (0–3)	Mean ± SD	0.33 ± 0.75	2.0 ± 1.22			<0.05 *

Pred, Prednisolone; Tac, Tacrolimus; CyA, Cyclosporine; MMF, Mycophenolate; Mz, Mizoribine; g, Glomerulitis; ptc, Peritubular capillaritis; N.S, no significant. Significant changes from baseline were evaluated using an analysis of variance and are indicated with asterisks (* *p* < 0.05, *** *p* < 0.001).
